# Striking Bone Marrow Plasmacytosis Resulting From Visceral Leishmaniasis

**DOI:** 10.1002/ajh.70368

**Published:** 2026-05-12

**Authors:** Vishakha Sovani, Jennifer Ryan, Barbara J. Bain

**Affiliations:** ^1^ Haematological Malignancy Diagnostics, Department of Cellular Pathology Nottingham University Hospitals NHS Trust Nottingham UK; ^2^ Faculty of Medicine, Centre for Haematology St Mary's Hospital Campus of Imperial College London UK

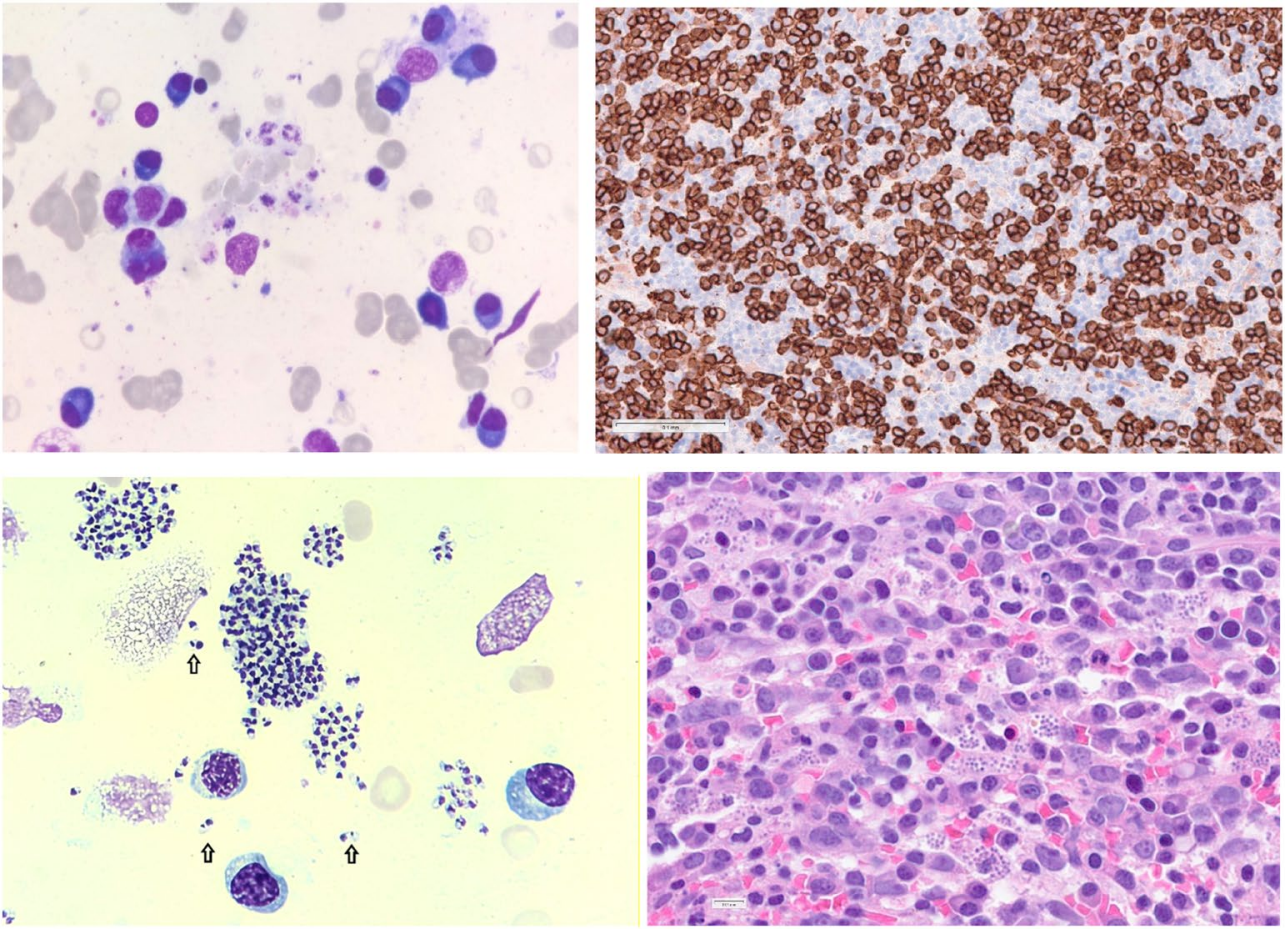

A 57‐year‐old UK national presenting to the emergency department following a fall revealed a history of 4 months of progressive constitutional symptoms: weight loss, recent ear infection, and intermittent epistaxis. He had resided in Spain from 2021 until returning to the UK 6 months before this presentation. On examination, he had cervical, axillary, and inguinal lymphadenopathy without palpable hepatosplenomegaly. Ulcerated plaques on the lower limbs prompted initial antimicrobial therapy for suspected cellulitis.

Laboratory investigations revealed pancytopenia (Hb 54 g/L, WBC 3.43 × 10^9^/L, platelets 46 × 10^9^/L) with marked rouleaux on blood film examination. There was marked hypoalbuminemia (10 g/L) and elevated immunoglobulin (Ig) G (89 g/L) with a serum free light chain kappa: lambda ratio of 1.58. Imaging showed splenomegaly (174 mm), multiple lung nodules (3–15 mm), bilateral pleural effusions, cardiomegaly, and widespread subcutaneous edema. Echocardiogram demonstrated a left ventricular ejection fraction of 38% with diastolic dysfunction.

A bone marrow (BM) aspirate and trephine biopsy sections revealed striking plasmacytosis accounting for approximately 60% of cellularity (top left, BM aspirate, May–Grünwald–Giemsa (MGG), ×50 objective; top right, trephine biopsy section, immunohistochemistry for CD138, ×20), a degree of infiltration that would typically raise serious concern for plasma cell neoplasia. However, plasma cells were cytologically normal, the serum Ig was polyclonal, and immunohistochemistry similarly showed no light chain restriction. The demonstration of a polytypic plasma cell population excluded a plasma cell neoplasm.

Critically, careful high‐power microscopic examination of both aspirate and trephine biopsy sections showed increased macrophages and revealed numerous intra‐ and extracellular *Leishmania* amastigotes (bottom left, BM aspirate, MGG ×100; bottom right, BM trephine biopsy section, hematoxylin, and eosin ×80). These organisms appear as 2–4 μm intracellular bodies with characteristic eccentric kinetoplasts (arrows in bottom left image). Without systematic high‐power examination of the macrophage population, these diagnostic organisms could easily be overlooked, particularly when presented against a background of marked plasmacytosis. Skin biopsy independently confirmed intra‐ and extracellular amastigotes within dermal macrophages, indicating cutaneous Leishmaniasis. Following completion of therapy with liposomal amphotericin B, the blood count normalized, skin lesions resolved, and an echocardiogram showed recovery of left ventricular function.

Such striking bone marrow plasmacytosis, which could be confused with a plasma cell neoplasm, is rare but can occur as a reaction to lymphoma, in chronic inflammatory conditions, in various infections, and during an adverse drug reaction. This case illustrates the diagnostic importance of bone marrow examination in pancytopenic patients from a region endemic for Leishmaniasis or with a relevant travel history. The striking combination of amastigote‐laden marrow with reactive plasmacytosis mimics hematological malignancy but carries distinctly different management implications. Prompt morphological recognition enables rapid infectious disease referral and appropriate antimicrobial therapy.

## Conflicts of Interest

The authors declare no conflicts of interest.

## Data Availability

The data that support the findings of this study are available from the corresponding author upon reasonable request.

